# Insights of Worsening Renal Function in Type 1 Cardiorenal Syndrome: From the Pathogenesis, Biomarkers to Treatment

**DOI:** 10.3389/fcvm.2021.760152

**Published:** 2021-12-14

**Authors:** Kang Fu, Yue Hu, Hui Zhang, Chen Wang, Zongwei Lin, Huixia Lu, Xiaoping Ji

**Affiliations:** The Key Laboratory of Cardiovascular Remodeling and Function Research, Chinese Ministry of Education, Chinese National Health Commission and Chinese Academy of Medical Sciences, The State and Shandong Province Joint Key Laboratory of Translational Cardiovascular Medicine, Qilu Hospital of Shandong University, Jinan, China

**Keywords:** worsening renal function, type 1 cardiorenal syndrome, acute heart failure, pathogenesis, biomarker, treatment

## Abstract

Type-1 cardiorenal syndrome refers to acute kidney injury induced by acute worsening cardiac function. Worsening renal function is a strong and independent predictive factor for poor prognosis. Currently, several problems of the type-1 cardiorenal syndrome have not been fully elucidated. The pathogenesis mechanism of renal dysfunction is unclear. Besides, the diagnostic efficiency, sensitivity, and specificity of the existing biomarkers are doubtful. Furthermore, the renal safety of the therapeutic strategies for acute heart failure (AHF) is still ambiguous. Based on these issues, we systematically summarized and depicted the research actualities and predicaments of the pathogenesis, diagnostic markers, and therapeutic strategies of worsening renal function in type-1 cardiorenal syndrome.

## Introduction

Acute heart failure (AHF), characterized by acute or subacute worsening symptoms and signs of heart failure (HF), is an intractable clinical and public health problem with high morbidity, mortality, and economic burden. AHF commonly coexists with numerous complications and renal dysfunction may be the most frequent one with a prevalence of approximately 25–40% (1, 2). To better apprehend the disorders of the coexistence of the concomitant impairment of both the cardiac and renal function, whereby acute or chronic dysfunction in one organ may induce acute or chronic dysfunction of the other, the terminology “cardiorenal syndrome” (CRS) is nominated (3). Depending upon the chief culprit of the pernicious and bidirectional insufficiency of both organs, CRS can be categorized into five clinical subtypes and acute worsening renal function (WRF) caused by the acute deterioration of cardiac function is termed as type-1 CRS (CRS-1 or acute CRS) (4, 5).

Impaired kidney function is an important independent prognostic factor for adverse events including cardiovascular mortality, the longer length of in-hospital stay, and HF re-hospitalization for AHF patients (6–8). Related clinical studies have validated that even an insignificant increase (as low as 0.2 mg/dL) in serum creatinine correlates with a poor prognosis for AHF patients (9). CRS-1 is a tremendous obstacle to nephrologists and cardiologists. However, there are several problems of WRF in CRS-1 that need to be solved. First, there is still a scarcity of a precise and quantitative description of WRF while the criteria of a 0.3–0.5 mg/dl rise in serum creatinine concentration, a 25% increase in plasm creatinine levels, or a decline in glomerular filtration rate (GFR) of 9−15 ml/min during AHF admission is established and has been accepted by some clinical trials (10–13). Second, the etiologies of WRF in CRS-1 remain unclear. Numerous factors, including hemodynamic imbalance, neurohormonal activation, and oxidative stress, may be involved in the pathogenesis. The exhaustive mechanism has not been absolutely elucidated. Third, while an increasing number of biomarkers have been explored and utilized in the diagnosis of renal dysfunction, the diagnostic efficiency, sensitivity, and specificity are still skeptical in the clinical practice. Last, renal safety of therapeutic strategies for AHF, such as decongestant therapy, is still debatable and some agents may be kidney toxic. Taking into account these controversies, WRF in CRS-1 is a noteworthy topic.

In this review, we summarized the latest findings of WRF in CRS-1, including the pathogenesis, clinical parameters facilitating diagnosis, and treatment strategies. A comprehensive and deep understanding of WRF in CRS-1 may provide new insights for early identification and effective treatment.

### Definition of WRF

Although the term WRF is commonly regarded as an acute and/or sub-acute change that occurs to kidney function, exhibiting an increase of serum creatinine concentration or a decline of the estimated glomerular filtration rate (eGFR) during AHF, a precise and unified definition of WRF in CRS-1 has not been given for that different renal injury biomarkers and different amplitude of variation which is considered significant were adopted in different studies. To tackle this issue, various definitions and criteria of WRF were proposed by three different consensus groups: Risk, Injury, Failure, Loss of kidney function, and End-stage kidney disease (RIFLE), Acute Kidney Injury Network (AKIN), and Kidney Disease: Improving Global Outcomes (KDIGO) (14–16). Different stages of acute kidney injuries (AKI) /WRF are classified in all sets of criteria, which are described in Table 1.

**Table 1:** Kidney Disease: Improving Global Outcomes (KDIGO), Acute Kidney Injury Network (AKIN), and Risk, Injury, Failure, Loss of kidney function, and End-stage kidney disease (RIFLE) criteria of AKI/WRF.

**Stage**	**Serum creatinine criteria**	**Urine output criteria**	**Annotation**
**KDIGO**			
1	1.5–1.9 times baseline or ≥0.3 mg/dl (≥26.5 μmol/L) increase	<0.5 ml/kg/h for 6–12 hours	The definition of AKI requires the increase in serum creatinine≥0.3 mg/dl within 48h or the change of serum creatinine ≥1.5 times within 7 days
2	2.0–2.9 times baseline	<0.5 ml/kg/h for ≥12 h	
3	≥3 times baseline or increase in serum creatinine to ≥4.0 mg/dl (≥353.6 μmol/L)	<0.3 ml/kg/h for ≥24 h or anuria for ≥12 h	
**AKIN**			
1	Increase in serum creatinine of ≥0.3 mg/dl (≥26.5 μmol/L) or increase to ≥150–200% (1.5- to 2.0-fold) from baseline	<0.5 ml/kg/h for 6–12 h	The definition requires an abrupt (within 48 h) decline in kidney function currently
2	Increase in serum creatinine to >200–300% (>2.0- to 3.0-fold) from baseline	<0.5 ml/kg/h for ≥12 h	
3	Increase in serum creatinine to >300% (>3.0-fold) from baseline or serum creatinine ≥4.0 mg/dl (≥353.6 μmol/L) with an acute rise of at least 0.5 mg/dl (44 μmol/L)	<0.3 ml/kg/h for ≥24 h or anuria for ≥12 h	
**RIFLE**			
Risk	Increase in serum creatinine × 1.5 times or GFR decrease >25%	<0.5 ml/kg/h for 6–12 h	Serum creatinine changes are abrupt (within 1–7 days), sustained for more than 24 h
Injury	Increase in serum creatinine × 2.0 times or GFR decrease >50%	<0.5 ml/kg/h for for 12–24 h	
Failure	Increase in serum creatinine × 3.0 times, GFR decrease > 75% or increase in serum creatinine to ≥4.0 mg/dl (≥353.6 μmol/L) with an acute rise >0.5 mg/dl (44 μmol/L)	<0.3 ml/kg/h for ≥24 h or anuria for ≥12 h	
Loss	Persistent acute renal failure = complete loss of kidney function >4 weeks	-	
End-stage kidney disease	End-stage kidney disease >3 months	-	

## Pathogenesis

**Figure 1:**
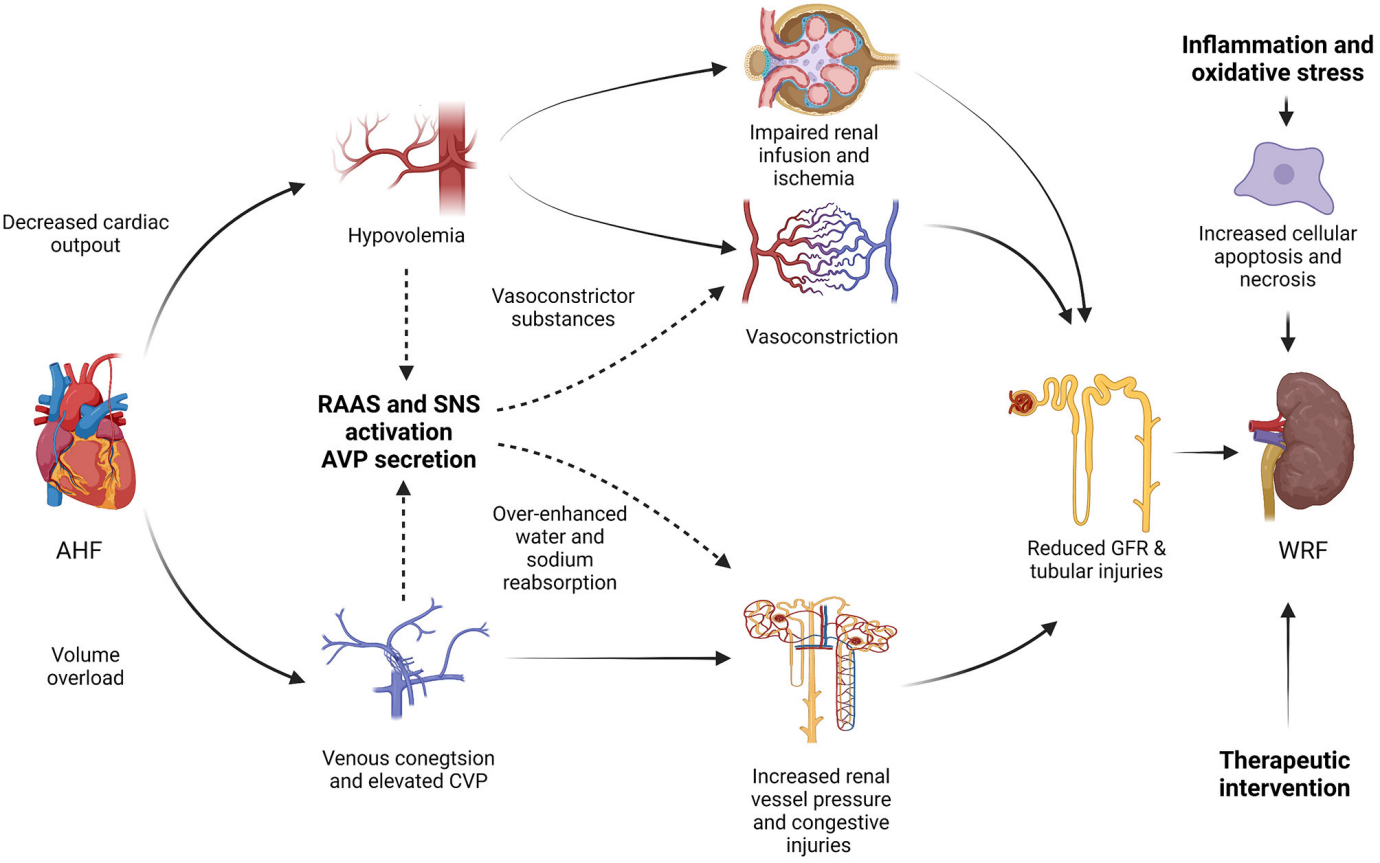
Pathogenesis mechanisms of worsening renal function (WRF) in type-1 cardiorenal syndrome (CRS-1). AHF, acute heart failure; RAAS, renin-angiotensin-aldosterone system; SNA, sympathetic nervous system; AVP, arginine vasopressin; CVP, central venous pressure; GFR, glomerular filtration rate; WRF, worsening renal function; CRS, cardiorenal syndrome.

The etiologies and pathogenic mechanisms of WRF in CRS-1 patients are multifactorial and ambiguous. The hemodynamic imbalance, neurohormonal activation, sympathetic activity, pharmacological interventions, inflammation along with oxidative stress may be involved in the pathogenesis of WRF in CRS-1 (Figure 1) (17).

### Hemodynamic Imbalance

The hypovolemia caused by diminished cardiac output or over-quick decongestion therapy can lead to impaired renal infusion. The reduced renal perfusion, or so-called impaired renal “preload”, is regarded as the dominating etiology of WRF in CRS-1 according to the historical mainstream viewpoints (18). Kidney artery under-filling due to hypovolemic status can lead to renal cortical ischemia or infarction. Besides, inadequate kidney perfusion can induce acute ischemic tubular necrosis (19). Hypovolemia can also activate the neurohormonal activation as described hereinafter. The vasoconstriction and sodium-retaining neurohormones, including angiotensin II and renin, will be over-generated and over-secreted as an auto-compensatory mechanism (20). However, this hypothesis is oversimplified and can only partially explain WRF in CRS-1, especially for patients with AHF with obviously impaired left ventricular systolic function and decreased cardiac output. For patients with normal left ventricular systolic function, videlicet, persevered left ventricular ejection fraction (LVEF), or for those with isolated right ventricular failure and elevated pulmonary artery pressure, the theory is unpersuasive (21). In fact, the proportion of WRF in CRS-1 caused by diminished cardiac output and reduced renal vasculature perfusion may be relatively low. The clinical manifestation of the majority is a “warm and wet” pattern, rather than a “cold” pattern, which refers to the presentation of preserved left ventricular systolic function and/or normal blood volume (22). The Evaluation Study of Congestive Heart Failure and Pulmonary Artery Catheterization Effectiveness (ESCAPE) trial also revealed no relationship between hemodynamic parameters and WRF in congestive heart failure patients receiving pulmonary artery catheter–guided therapy (23). On this occasion, “forward failure” caused by low cardiac output should not be regarded as the main determinant of renal dysfunction in CRS-1 patients and renal congestion may play a more vital role.

Renal congestion induced by systemic congestion and volume overload may also participate in the pathogenesis (24). For patients with a “warm and wet” pattern, systemic congestion, increased pulmonary and/or renal congestion are the main hemodynamic profile. The exact mechanism of WRF induced by renal congestion has not been well clarified. Increased renal vessel pressure caused by renal congestion may trigger interstitial edema, tubular dysfunction, and reversible azotemia (25, 26). The phenomenon that increased central venous pressure (CVP) and/or raised intra-abdominal pressure (IAP) positively correlate with WRF in AHF also provides more testimonies. Previous literature has indicated that the increase in CVP has a strong association with declined GFR and WRF in patients with AHF (27). Mullens et al. demonstrated that it is the elevation of the admission baseline CVP, mean CVP, and discharge CVP rather than the cardiac index (CI) or other hemodynamic parameters that are strongly correlated with the increased risk of WRF for patients with acute decompensated heart failure (ADHF) (28). The role of CVP in maintaining renal perfusion pressure and trans-renal perfusion is decisive. For patients with systemic congestion, CVP elevates dramatically. Renal interstitial pressure can escalate along with increased CVP which may cause the congestion of renal vasculature, thus congestive kidney failure is developed. Analogous to mechanisms of liver failure caused by hepatic congestion, the congestive kidney injuries, manifesting as ischemic injury of renal parenchyma and hypoxia damage of renal cortex, will emerge under the increased renal interstitial pressure (12, 29). Raised IAP also contributes to the development of WRF. Although distinct ascites can be only detected in a small population of patients with acute CRS, symptoms of visceral/tissue edema are prevalent for patients with systemic congestion and the prevalence of raised IAP can reach up to 60% in patients ADHF. On one hand, elevated IAP indirectly increases CVP and may further induce congestive renal failure. On the other hand, the kidney is compressed when exposed to raised IAP and renal infusion displays a precipitous decline. Hence ischemic injuries will develop due to the reduced renal blood flow. Mullens et al. also illustrated that elevated IAP was associated with WRF for patients with ADHF and reductions of IAP were associated with improved renal function (30).

### Neurohormonal Activation

The renin-angiotensin-aldosterone system (RAAS) is crucial in maintaining the hemodynamic homeostasis and plays a major role in the cardiorenal physiological/pathophysiologic bidirectional interaction in CRS-1 (31). The persistent renal hypoperfusion results in RAAS overactivation. Besides, sympathetic nerve activity (SNA) and the secretion and release of arginine vasopressin (AVP) will also be increased to tackle systemic hypovolemia and to maintain kidney blood flow.

The activation of RAAS plays a vital role in maintaining systemic and renal hemodynamic hemostasis and contributes to cellular hypertrophy, apoptosis, and fibrosis in both the heart and the kidney. At the cardiac level, it can reflect the degree of cardiac remodeling, ventricular hypertrophy, and fibrosis. What is more, it promotes sodium and water retention, mediates the redistribution of intrarenal perfusion which refers to the reduced medullary blood flow and increased cortical blood flow, to compensate for the hemodynamic changes in CRS-1 (32). In detail, angiotensin II, renin, as well as numerous other vasoactive agents, including vasodilators such as prostaglandin I2 and nitric oxide, are subsequently generated in response to the activation of RAAS. The former, vasoconstrictors, can constrict efferent arterioles, and the latter, vasodilators, can dilate afferent arterioles. Thus the glomerular hydrostatic pressure and filtration are preserved (22). In this compensated stage, the kidney function is approximately normal. With the progression of WRF, both afferent and efferent arterioles are both constricted, thus the vasoconstriction effect of angiotensin II dominates renal hemodynamic physiopathologic changes. In the decompensated phase, GFR falls dramatically (33, 34).

Overactivation of RAAS accounts for approximately 50% of the over-production of aldosterone (35). The mineralocorticoid, aldosterone, is of great importance in increasing sodium and water retention and the regulation of blood pressure and fluid homeostasis. Furthermore, Angiotensin II and aldosterone are tightly associated with the hypertrophy, apoptosis, and fibrosis of renal tubular cells (36). On one hand, Angiotensin II and aldosterone promotes the expression of numerous profibrotic molecules, such as plasminogen activator inhibitor 1, osteopontin, and galectin-3, which promote collagen and matrix production. On the other hand, angiotensin II and aldosterone also stimulates mitochondrial production of reactive oxygen species (ROS) and thus exacerbates renal tubulointerstitial fibrosis and endothelial dysfunction through the inflammatory pathway (37).

The SNA will also be potentiated for hypovolemia and decreased artery pressure (38, 39). Angiotensin is the stimulator of the sympathetic nervous system (SNS) and can also contribute to the activation of SNS *via* numerous biological effects, such as the direct activation of critical sympathoexcitatory neurons in the paraventricular nucleus of the hypothalamus (40). At the cardiac level, it increases cardiac output while worsens cardiac fibrosis and hypertrophy. For the kidney, over-activated SNS stimulates not only α1-adrenergic receptors on vascular smooth muscle cells to enhance vasoconstriction but also β1-adrenergic receptors on juxtaglomerular cells to increase the secretion of renin, which can activate RAAS in reverse (41). Hence, enhanced SNA results in vessel constriction, increased systemic vascular resistance, and sodium retention.

Arginine vasopressin (AVP), the antidiuretic hormone secreted by the posterior pituitary gland receiving the stimulation of hypovolemia and increased osmolality, also takes on pivotal importance in sustaining the renal filtration fraction (42). AVP can activate V1 receptors on the vasculature, which results in renal vessels constriction and elevated vascular resistance. V2 receptors on principal cells can also be activated thus water and sodium reabsorption, as well as hyponatremia, is aggravated (43). Both water and sodium-retaining neurohormones work together to maintain hemodynamic homeostasis and sufficient kidney perfusion pressure. Hypervolemia induced by water and sodium retention also triggers congestive renal injuries.

### Inflammation and Oxidative Stress

The increase of proinflammatory cytokines and oxidative stress is the vital factor for the pathogenesis of WRF, which have been validated by numerous *in vivo* and *in vitro* experimental studies (44). The underlying mechanisms may incorporate oxidative stress and inflammation leading to monocyte phenotype transition, kidney interstitial fibrosis, and renal cellular apoptosis. Inflammatory and apoptosis pathway plays a crucial role in the pathogenesis of WRF in CRS-1. Inflammatory cytokines can induce AKI *via* activating death signaling receptors and other various signaling pathways. It can cause endothelial function impairment, renal vasoconstriction, and capillary obstruction (45, 46). The oxidative stress pathway is a classic pathway inducing cellular injury, interstitial fibrosis, and organ dysfunction. An increase of oxidative stress leads to the over-generation of ROS and reactive nitrogen species (RNS), which facilitates the formation of proinflammatory and profibrotic milieu. The over-activation of RAAS can also stimulate the over-production of ROS and RNS in the kidney (47). Furthermore, over-production of ROS and RNS exacerbates renal and cardiovascular structural and functional abnormalities through inactivating mitochondrial enzymes, accelerating DNA damage, and promoting base hydroxylation (46). Both cardiac and renal injuries are thus developed.

The inflammatory and oxidative stress activities are increased in acute CRS. *Vitro* experiments conducted by Pastori et al. revealed that when adding the plasm of patients with acute CRS, the apoptosis of monocyte cell lines was obviously upregulated vs. those incubated with plasm from patients with AHF or healthy individuals. Besides, the expression levels of caspase-3 and-8 of monocytes were in line with apoptosis rates. Plasm concentrations of inflammatory cytokines including Interleukin (IL) −6 and −18 were also apparently higher in patients with acute CRS than which in patients with AHF (48). A similar phenomenon, which refers to the more obvious elevation of plasm levels of inflammatory cytokine and markers of oxidative stress including IL-6 and myeloperoxidase (MPO) in acute CRS patients compared with patients with AHF, was also observed in the study of Virzì et al. (46, 49). *Vivo* experiments also showed significant elevation of serum concentration of cytokines and MPO in patients with CRS-1 vs. patients with AHF without renal insufficiency (50). An observational pilot study reflected that the activation of peripheral blood monocytes may be the stimulator of inflammatory pathway way in acute CRS patients. Monocyte phenotype transition and apoptosis are crucial in the pathogenesis of WRF (51).

## Markers for WRF

### Creatinine

Creatinine is the most widely used biomarker to evaluate GFR. As the pioneer of biomarkers estimating renal function, creatinine negatively correlates with GFR. Creatinine is a 113kDa end-product of creatine phosphate metabolism. Creatine is a nitrogenous organic acid that is predominantly generated in the kidney and liver and is mainly transported to and metabolized in skeletal and heart muscles tissues for energy generation. Serum creatinine is generated during the spontaneous, non-enzymatic anhydration of creatine in muscle cells (62, 63). Creatinine is filtered by the glomerulus. The highlighted peculiarity of creatinine is that it will not be reabsorbed by the renal tubules (64). Since creatinine was discovered, it has been wildly used as the “golden standard” to estimate GFR in clinical practice.

There are blemishes of creatinine in assessing renal function. Above all, the synthesis and secretion of creatinine are heterogeneous across individuals. The production of creatine principally depends on muscle mass, physical activity, dietary meat consumption, ethnic and age factors. Chronic illness, including inflammatory disease and malignant tumors, can also decrease creatinine generation. For another, the clearance of creatinine is also influenced by diversified factors such as pharmacotherapy including cimetidine and trimethoprim (65). Moreover, the relationship between creatinine and GFR is non-linear and exponential (66). On some occasions, creatinine is impotent to detect a mild alternation in GFR and will underestimate the degree of kidney impairment. Considerable reduction of GFR may accompany the weeny elevation of serum creatinine concentration, especially in patients with advanced age and low muscle mass (8). In addition, the uplift of serum creatinine is comparatively lagged behind AKI and is insensitive to tubular impairment (67). These characteristics impose restrictions on the utilization of creatinine.

### Cystatin C

Cystatin C (CysC), a non-glycosylated 13kDa protein, is a member of endogenous cysteine proteinase inhibitors (68). Cystatin C is synthesized in all human nucleated cells and is encoded by the CST3 gene, a housekeeping gene located in chromosome 20 (69). For this reason, the generation rate of CysC is relatively constant. Similar to creatinine, CysC is freely filtered by glomeruli (70). CysC is completely reabsorbed and metabolized by renal tubular epithelial cells. The changing trend of serum concentration of CysC is in keeping with which of GFR, thus CysC is considered as a novel and dependable biomarker in identifying kidney disease (71). For that, CysC is insensitive to various confound factors, numerous studies have indicated that the performance of CysC in diagnosis renal insufficiency is comparably superior to creatinine (68). Rafouli-Stergiou et al. revealed that a rise in cystatin C during the admission of patients with ADHF was an independent predictor of short-term prognosis. Besides, the combination of cystatin C and NT-proBNP could provide additional prognostic information for WRF in CRS-1 (66). One multicenter clinical study revealed that CysC could well predict AKI in the admission patients with AHF and a rise in cystatin C > >0.3 mg/L within 48h from admission positively correlated with higher in-hospital mortality and longer hospital stay (Table 2) (52).

**Table 2:** Summary of studies on biomarkers of WRF in CRS-1 in this review.

**Study**	**Study population**	**Biomarker**	**Timing of biomarker evaluation**	**Conclusion**	**Sensitivity/specificity**
Lassus et al. (52)	292 patients with AHF	Plasm cystatin C	48h after admission	Cystatin C > 0.3 mg/L could predict WRF in AHF.	AUC: 0.92 Specificity: 90% sensitivity: 77%
Chen et al. (53)	732 patients with ADHF	Urinary NGAL	Every 24 h for the first 7 days during hospitalization	Urinary NGAL facilitated the identification of progressive AKI for ADHF patients.	AUC: 0.74 (95% CI 0.67–0.82) Specificity: 63% sensitivity: 76%
Murray et al. (54)	930 patients with AHF who have been treated or with plan to treat with IV diuretics	Urinary NGAL	Day of enrollment within 2 h of first IV diuretic dose; 2 to 6 h later; hospital days 1, 2 and 3; and day of discharge or anticipated discharge	Diagnostic value of urinary NGAL was limited. Urinary NGAL was not superior to creatinine for predicting WRF for AHF patients.	AUC of the peak urinary NGAL: 0.51 AUC of the first urinary NGAL: 0.61
Okubo et al. (55)	138 patients with AHF	Urinary L-FABP	First day of hospital admission	An increased urinary L-FABP level may predict WRF for AHF patients.	Urinary L-FABP level ≥ 8.4 μg/g creatinine was independently associated with WRF (HR 1.8, *p* = 0.01)
Legrand et al. (56)	87 patients with ADHF	Urinary KIM-1, NAG	Unclear	Urine biomarkers of renal injury (including KIM-1 and NAG) did not predict WRF.	AUC of KIM-1: 0.49 (95% CI 0.37–0.62) AUC of NAG: 0.46 (95% CI 0.31–0.61)
Ahmad et al. (57)	283 patients with ADHF and pre-existing renal dysfunction	Urinary KIM-1, NGAL and NAG	Daily for the 72-h study intervention period	Tubular injury biomarker levels did not differ between patients with and without WRF defined by cystatin C.	72-h changes in NGAL, KIM-1 and NAG between patients with and without WRF did not reach statistically significance (*p*-value was respectively 0.21, 0.22 and 0.46)
Sokolski et al. (58)	132 patients with AHF	Urinary KIM-1 and urinary NGAL	Every 24 h for the first 3 days during hospitalization	Urinary NGAL and urinary KIM-1 may predicate the development of WRF in AHF.	AUC of baseline urinary NGAL: 0.76 (95% CI 0.63–0.90) AUC of urinary NGAL at day 2: 0.83 (95% CI 0.73–0.93) AUC of urinary NGAL at day 3: 0.77 (95% CI 0.60–0.94) AUC of urinary KIM-1 at day 2: 0.74 (95% CI 0.59–0.90)
Funabashi et al. (59)	708 patients with AHF	Urinary NAG	First day of hospital admission	Urinary NAG did not corelate with renal function.	In multivariable linear regression analyses, β- coefficient = 0.005, *p* = 0.93
Virzì et al. (49)	80 patients with AHF	Plasm IL-18	Within 8 h of hospital admission	IL-18 was higher in CRS type 1 compared with AHF patients.	Difference of plasm IL-18 concentration in AHF patients with and without WRF was meaningful (*p* <0.001)
Atici et al. (60)	111 patients with ADHF	Urinary KIM-1, TIMP-2 and IGFBP-7	Unclear	Urinary [TIMP-2]·[IGFBP7] could predict WRF, while the diagnostic value of urinary KIM-1 was mild.	AUC of urinary [TIMP-2]·[IGFBP7]: 0.75 (95% CI 0.61–0.88) AUC of urinary KIM-1: 0.54 (95% CI 0.37–0.70)
Schanz et al. (61)	40 patients with ADHF	Urinary TIMP-2 and IGFBP-7	First day of enrollment and daily thereafter	Urinary [TIMP-2]·[IGFBP7] could discriminate for AKI stage 2–3 in ADHF.	AUC of samples collected within 24 h of enrollment: 0.84 (95% CI: 0.72–0.93) At the 0.3 cutoff for [TIMP-2]·[IGFBP7], specificity: 73% sensitivity: 86% AUC of samples collected over 7 days: 0.77 (95% CI: 0.65–0.88)

*AHF, acute heart failure; CRS, cardiorenal syndrome; AUC, area under the curve; ADHF, acute decompensated heart failure; NGAL, neutrophil gelatinase-associated lipocalin; 95%CI, 95% confidence interval; AKI, acute kidney injury; WRF, worsening renal function; L-FABP, liver-type fatty acid-binding protein; HR, hazard rate; KIM-1, kidney injury molecule-1; NAG, N-acetyl-β-(D)-glucosaminidase; IL-18, Interleukin-18; CRS, cardiorenal syndrome; IGFBP-7, insulin-like growth factor-binding protein 7; TIMP2, tissue inhibitor of metalloproteinase-2*.

### Neutrophil Gelatinase-Associated Lipocalin

As a member of the lipocalin family, neutrophil gelatinase-associated lipocalin (NGAL) is a 25kDa protein, which is filtered by the glomerulus and is immediately reabsorbed in proximal tubule through a megalin-dependent pathway (72). The serum concentration of NGAL is relatively low in healthy individuals, whereas its plasm level significantly elevates (about 300-folds) in response to tubular epithelial damage (73). The expression of NGAL is upregulated in kidney tissue when AKI is developed, which may result in the increase of NGLA plasm concentrations. Furthermore, impaired reabsorption function of the proximal tubule may contribute to the increased levels in urine (74). Previous studies have validated the efficiency and accuracy of urinary NGAL in the diagnosis of AKI and WRF in patients with AHF, while the sensitivity and specificity of plasm NGAL are not so excellent (53). Interestingly, the Acute Kidney Injury N-gal Evaluation of Symptomatic heart failure Study (AKINESIS) study confirmed that the diagnostic value and prognostic predicting value of plasm NGAL for WRF in patients with AHF was limited and was not superior to creatinine. Similarly, the performance of urine NGAL in predicting WRF and prognosis did not overmatch creatinine (54).

### Liver-Type Fatty Acid-Binding Protein

Fatty acid-binding protein (FABP) is a 14KDa molecule protein and belongs to the superfamily of lipid-binding proteins. FABP plays a significantly important role in the metabolism and transportation of fatty acids (75). FABP consists of nine tissue-specific subtypes, including liver (L), intestinal (I), muscle and heart (H), adipocyte (A), epidermal (E), ileal (IL), brain (B), myelin (M), and testis (T) (76). For liver-type fatty acid-binding protein (L-FABP), it is expressed in both hepatocytes and tubular epithelial cells. It participates in the transportation of fatty acids and is essential to fatty acid β-oxidation and power supply of tubular epithelial cells. For patients with AKI, increased oxidative stress promotes the over-dosed production of ROS. For the peroxidation of plasma membrane and cytoplasmic membrane mediated by ROS, the cytotoxic lipid peroxidation products, which are bound and cleared by L-FABP, are excessively generated and accumulated in proximal tubules. Thus L-FABP was excreted from the proximal tubules into urine together with cytotoxic lipids. Urinary L-FABP has been proposed as an emerging acute proximal tubules injury biomarker for WRF in CRS-1 despites few related studies (77, 78). Yousaku Okubo et al. revealed that urinary L-FABP can predict WRF in patients with AHF and patients with higher L-FABP levels were more likely to have rehospitalization (55).

### Kidney Injury Molecule-1

Kidney injury molecule-1 (KIM-1) is a 38.7kDa transmembrane glycoprotein, which is a member of type I transmembrane glycoproteins (79). KIM-1 is principally expressed at the apical membrane of proximal tubular epithelial cells and its expression maintains a low state under normal circumstances. The expression of KIM-1 is dramatically upregulated when the renal tubule undergoes different injuries including ischemia-reperfusion injury (80, 81). KIM-1 is regarded as a promising biomarker for detecting renal tubular injuries, while its sensitivity and accuracy in the diagnosis of AKI have not been validated (82). Studies engaged in the association between KIM-1 and WRF in patients with CRS-1 are rare. Legrand et al. revealed a slight but non-statistically significant association between KIM-1 and increased risk of WRF for AHF patients in the Biomonitoring and Cardiorenal Syndrome in Heart Failure Trial (BIONICS) trial population (56). A similar result was drawn in the Renal Optimization Strategies Evaluation AHF (ROSE-AHF) trial, which exhibited no relationship between the change of plasm KIM-1 level and the occurrence of WRF in patients with AHF (57). On the contrary, urinary KIM-1 is a meaningful biomarker for predicting WRF. Sokolski et al. exhibited the excellent diagnostic value of urinary KIM-1 for WRF (58).

### N-Acetyl-β-(D)-Glucosaminidase

N-acetyl-β-(D)-glucosaminidase (NAG) is a brush-border lysosomal enzyme found in several human cells including proximal tubule cells (83). On the grounds that NAG is a large molecular weight compound (>>130kDa), NAG obviates the elimination through glomerular filtration and thus, the elevation of urinary NAG levels can be considered as a tubular origin (84). Urinary NAG is regarded as an outstanding predictor reflecting the impairment of tubular injury. Funabashi et al. demonstrated that for inpatients with AHF recruited from the National Cerebral and Cardiovascular Center Acute Decompensated Heart Failure (NaDEF) registry study, those with higher urinary NAG tended to have lower eGFR than those with low urinary NAG levels, while multivariable linear regression analyses showed no significant relation between renal function and urinary NAG concentrations. Besides, elevated urinary NAG levels correlated with long-term adverse events (59). In contrast, the ROSE-AHF trial indicated no relationship between the development of WRF and the increase of urinary NAG (57). Related clinical studies are relatively limited and further studies should be conducted.

### Interleukin-18

As a member of the IL-1 family, Interleukin-18 (IL-18), which was first described as an “interferon (INF) γ-inducing factor,” is an 18kDa biologically active proinflammatory cytokine (85). IL-18 is involved in numerous renal pathogenic processes such as apoptosis, ischemia-reperfusion injuries, allograft rejection, and malignancy. IL-18 is generated by proximal tubules and is excreted into the urine after acute ischemic injuries, which have been verified by several *in vivo* studies (86, 87). As mentioned above, inflammation and oxidative stress factors are crucial for the pathogenesis process of WRF. Hence inflammation cytokines (such as IL-18) may be potential candidates for the identification of WRF in the early stages of acute renal insufficiency. Parikh et al. revealed that urinary IL-18 could accurately predict WRF even in the first 24 h of onset of deterioration of renal function, which was obviously earlier than the elevation of serum creatinine (88). The Systolic Blood Pressure Intervention Trial (SPRINT) trial also indicated that IL-18 was an independent predictor of future risk of AKI with high sensitivity and accuracy (89). Virzì et al. indicated that plasma levels of proinflammatory cytokines including IL-18 were higher in CRS-1 patients compared with AHF patients, which hinted at the potential of IL-18 in the diagnosis of WRF in CRS-1 (49).

### Insulin-Like Growth Factor-Binding Protein 7 (IGFBP-7) and Tissue Inhibitors of Metalloproteinase-2 (TIMP2)

Insulin-like growth factor-binding protein 7 (IGFBP-7) and TIMP2 are both small molecular weight proteins (29 kDa and 24 kDa, respectively) and stimulators of G1 cell cycle arrest which involves in the pathogenesis of AKI (90, 91). Both biomarkers prevent the division of injured cells with damaged DNA. The normal cell cycle will re-initiate until the repair is accomplished. However, pathological changes such as senescence and fibrosis will emerge if the arrested cell cycle lasts for a too long period (92). These two biomarkers are both expressed, generated, and secreted in renal tubular cells at the early phase of AKI/WRF. Related studies have validated that the combination of these two biomarkers, which refers to urinary [TIMP-2]·[IGFBP7], had a significantly superior performance in the early identification and diagnosis of AKI compared with classic renal injury biomarkers such as KIM-1 (93, 94). When focused on its application in the diagnosis of WRF in CRS-1, associated studies are limited. In Atici et al.'s study, the levels of urinary [TIMP-2]·[IGFBP7] were significantly elevated in patients with acute CRS compared with those with simple AHF (60). Schanz et al. also revealed that urinary [TIMP-2]·[IGFBP7] was a promising biomarker in the early discrimination of WRF in patients with ADHF with considerable sensitivity and specificity. Furthermore, the urinary [TIMP-2]·[IGFBP7] also well predicted all-cause mortality at 1 year after discharge (61).

### B-Type Natriuretic Peptide (BNP)

B-type natriuretic peptide (BNP) and N-terminal proB-type natriuretic peptide (NT-proBNP) are well-established biomarkers for HF and have been universally manipulated in estimating the presence, severity, and prognosis of HF (95). The serum concentration of NT-proBNP can be influenced by a variety of factors, such as renal function, advanced age, blood pressure, severe infection, gender, and obesity (5). Hence BNP and NT-proBNP are not regarded as eligible and reliable biomarkers for the identification of WRF. Based on that, the term “the estimated mature BNP” (emBNP), which was calculated by subtracting proBNP from total BNP, has been proposed. It may contribute to the identification of WRF in CRS-1 patients for its peculiar clearing mechanisms (96). The emBNP is cleared through membrane-bound natriuretic peptide receptors A and C rather than kidney, whereas NT-proBNP was mainly cleared by renal excretion (97). Previous literature validated that patients with AHF and WRF had lower emBNP levels and higher NT-proBNP/emBNP ratios. Besides, NT-proBNP/emBNP ratios were associated with composite clinical events, including all causes of death and rehospitalization for HF (96).

## Treatment

### Diuretics

Congestion is the hallmark of AHF and decongestion therapy, especially diuresis pharmacotherapy, is the cornerstone for patients with AHF. However, controversies concerning the utilization of diuretics in patients with CRS-1 have never been eliminated for the reason that prior studies have identified the association between aggressive diuresis (loop diuretics in especial) and increased risk of WRF. The possibility that administration of diuretics may induce or aggravate WRF in AHF patients astricts the appropriate use of diuretics. The optimal diuretics therapeutic regimen in CRS-1 patients is still unclear.

It is a conundrum whether aggressive fluid removal *via* escalating doses of diuretics can be safely and effectively applied in the setting of AHF. It deserves the deliberateness to weigh up the pros and cons. From pathogenesis, diuretics therapy will rapidly mitigate congestion and reduce increased CVP, which may ameliorate the kidney dysfunction caused by congestive kidney damages. While hypovolemia and impaired renal preload will induce hypo-infusion renal injuries. Hence different viewpoints of rational diuretics use have been concluded in different clinical researches. The Description de la Filière de Soins dans les Syndromes d'Insuffisance Cardiaque Aigue (DeFSSICA) study indicated no limitation of loop diuretics in patients with AHF with renal insufficiency for the reason that the prognosis of patients with acute CRS on diuretics therapy was indiscriminate compared with that of patients with AHF (98). Conversely, the Randomized Evaluation of Heart Failure with Preserved Ejection Fraction Patients with Acute Heart Failure and Dopamine (ROPA-DOP) study and the DOSE-AHF study clarified the association between the utilization of diuretics and WRF in patients with AHF (99, 100). The DOSE-AHF study illustrated that patients with AHF receiving high-dose furosemide treatment were more vulnerable to transient WRF than those accepting low-dose furosemide (99). The ROPA-DOP study also revealed that a continuous infusion diuretic strategy was associated with a higher incidence of WRF in patients with heart failure with preserved ejection fraction (HFpEF) hospitalized for AHF treatment (100).

Given this evidence, how to suitably and optimally use diuretics is still a predicament. The dosage and mode of administration of diuretics are relatively crucial to balance the therapeutic effect of decongestion and the possibility of WRF. For the issue of administration mode, in Palazzuoli et al.'s study, patients receiving continuous furosemide infusion therapy were more vulnerable to WRF than those receiving bolus injections of furosemide, while the efficiency of diuresis was better in the former (101, 102). For the question of diuretics dosage, the DOSE-AHF study demonstrated that AHF patients on high-dose furosemide therapy had an increased risk of transient WRF vs. those on low-dose furosemide, and the primary endpoints (the patient's global assessment of symptoms) of two groups had non-significant difference (99). Carbohydrate antigen 125 (CA-125) diuretic-guided treatment proposed by Núñez et al. perhaps can solve the dilemma for that CA-125 is an excellent marker reflecting congestion. In this study, during the diuretic therapy process, serum CA-125 was detected and the dosage of diuretics would be determined and adjusted according to the plasm CA-125 concentration stratification. Individualized decongestion treatment may be realized when CA-125 diuretic-guided treatment is utilized.

### Vasopressin Antagonists

In the pathogenetic process of WRF in CRS-1, as described above, AVP is over-generated and over-secreted in response to neurohormonal activation. AVP can activate both V1 receptors on the vasculature and V2 receptors on principal cells resulting in renal vasculature vasoconstriction and the enhancement of water and sodium reabsorption (103). Tolvaptan, a selective V2 receptor antagonist of AVP, can act on the distal portion of the nephron and competitively blocks the bond of AVP and V2 receptors, which facilitates the activation of the aquaporin system and the prevention of water and sodium reabsorption (104).

The Efficacy of Vasopressin Antagonism in Heart Failure Outcome Study With Tolvaptan (EVEREST) trial has ascertained the efficacy and safety of tolvaptan in AHF treatment while the renal function was not included as observation criteria or endpoint criteria in this trial (105). Subsequent studies supplemented related evidence. The Clinical Effectiveness of Tolvaptan in Patients with Acute Heart Failure and Renal Dysfunction (AQUAMARINE) study validated that for patients with CRS-1, tolvaptan had better diuresis effectiveness compared with conventional diuretic therapy while the incidence of WRF was comparable (106). A secondary analysis of the AQUAMARINE study also revealed that patients with CRS-1 were well-tolerated to tolvaptan and tolvaptan can improve diuretic response (107). Another study conducted by Matsue et al. illustrated that for patients with AHF with renal dysfunction, tolvaptan could improve the prognosis and reduce the risk of all-cause death and HF readmission in specific individuals whose eGFR was 30mL/min/1.73m^2^ or above (108). All in all, vasopressin antagonist is an effective decongestion therapy with less influence on renal function.

### Vasodilators

Vasodilators are another fundamental therapeutic regimen and are the second most commonly used drugs for AHF treatment, which has been validated by current clinical practice guidelines. Vasodilators can rapidly reduce ventricular filling pressure and central venous tone, thus reducing both cardiac preload and afterload, and decreasing the myocardial oxygen consumption (109). In hypertensive AHF, vasodilators are widely used and their efficacy is considerable, while for patients with low systolic pressure and/or systemic hypotension, vasodilators should be avoided. For hemodynamic influences of vasodilators, which may induce WRF, the utilization of vasodilators in patients with AHF with renal dysfunction should be cautious.

Nesiritide, or recombinant human BNP, is a type of vasodilator with both natriuretic and diuretic effects. Nesiritide has the same amino acid sequence and pharmacological effect on endogenous BNP. It can interact with natriuretic peptide receptor A on vascular smooth muscle cells and endothelial cells and can activate the guanylyl cyclase pathway, resulting in an increase of intracellular cyclic guanosine monophosphate (110). Besides, it can antagonize the effect of RAAS. Nesiritide has been approved for the treatment of AHF and previous literature indicated that nesiritide can mitigate congestion with no influence on renal function. The Acute Study of Clinical Effectiveness of Nesiritide in Decompensated Heart Failure (ASCEND-HF) study revealed that compared with placebo, nesiritide did not improve clinical outcomes and did not worsen renal function (111, 112). The ROSE-AHF study also showed similar results. These randomized clinical trials validated the renal safety of nesiritide despite its feeblish effects on decongestion and improvement of clinical outcomes and prognosis.

Relaxin, a 6kDa hormone of pregnancy mainly secreted by the corpus luteum of the ovary, plays an important role in maintaining the homeostasis of cardiovascular and hemodynamic during pregnancy. Relaxin can cause systemic and renal vasodilation *via* stimulating the relaxin family peptide receptors (RXFP), which are widely distributed in the heart, skeletal muscle, kidney, arteries, veins, and various tissues and organs. The activation of RXFP will increase the generation of second messengers, thus activating the nitric oxide pathway and cyclic adenosine monophosphate (cAMP) pathway, promoting the production of vasodilator substances, resulting in decreased systemic and renal vascular tone (113, 114). Serelaxin, the recombinant human relaxin-2, has similar pharmacologic action to relaxin and is considered as a potential therapeutic agent for AHF (115). The RELAX-AHF study recruited AHF participants with mild-to-moderate renal dysfunction (with eGFR of 30–75 mL/min/1·73 m^2^) who would be randomly assigned to the serelaxin treatment group and placebo treatment group. The study revealed that serelaxin was associated with greater dyspnoea relief, reduced worsening HF events, reduced cardiovascular and all-cause mortality compared to placebo, while lower proportions of patients on serelaxin therapy had development of WRF and adverse events related to renal function impairment compared with the placebo group (116). Additional studies concerning relaxin/ serelaxin have been ongoing. In the Pre-relaxin for the treatment of patients with acute heart failure (RELAX-AHF) study, patients with AHF receiving intravenous relaxin therapy tended to have a similar risk of WRF vs. those on placebo therapy (117, 118). The RELAX-AHF-EU study explored the effect of serelaxin when standard-of-care (SoC) therapy was added in AHF patients with mild-to-moderate renal dysfunction (119). Patients receiving serelaxin+SoC therapy were less likely to suffer renal deterioration compared to those with SoC therapy alone. The RELAX-AHF-2 study also affirmed the renal safety of serelaxin (120). To sum up, serelaxin is beneficial to ameliorate congestion symptoms and signs and improve the prognosis of CRS-1 patients with potential kidney protective effects.

### Inotropes

For patients with AHF with low cardiac output and hypotension, especially for those with low systolic blood pressure, inadequate peripheral infusion, and poor response to strand therapy, inotropes are still effective remedies to maintain vital organs (including kidney) perfusion and function (121). Various studies have reported the renal protective effect of inotropes for AHF patients.

Dopamine, the most commonly used inotropes, can increase cardiac output by reducing cardiac afterload and may improve renal perfusion and GFR through dilating both afferent and efferent arterioles. However, only small-scale studies have validated the potential mechanism (122). The Dopamine in Acute Decompensated Heart Failure (DAD-HF) trial confirmed for AHF patients on high dose furosemide (20mg/h continuous for 8 h), the incidence of WRF was obviously higher than those on low dose furosemide combined with low dose dopamine (furosemide 5 mg/h plus dopamine 5 mg·kg^−1^·min^−1^ continuous infusion for 8 h). while there was no obvious difference in the 60-day mortality and/or rehospitalization rates between the two groups (123). In the DAD-HFIItrail, compared with the DAD-HFItrail, an isolated low dose furosemide (furosemide 5 mg/h continuous for 8 h) treatment group was added. Similar to the DAD-HFItrail, the incidence of WRF was higher in the high-dose furosemide group than the low-dose furosemide plus low-dose dopamine group and low-dose furosemide group at 24 h after initiation of treatment, no better prognosis was detected in latter groups during 1-year follow up period (124). Besides, controversial outcomes were concluded in other clinical trials including the ROSE-AHF study, the adrenergic inotropes exhibited more adverse events and no renoprotective effect compared with other types of inotropes such as levosimendan.

Levosimendan exerts its inotropic action through increasing sensitivity of troponin C to calcium in cardiomyocytes *via* c-AMP independent effect. Besides, it owns vasodilation effect through acting on adenosine triphosphate-sensitive potassium channels (K_ATP_ channels) in the smooth muscle cell. Based on these properties, levosimendan can dilate afferent arterioles of the kidney and ameliorate renal perfusion, thus increasing GFR. Some studies also indicated can selectively dilate afferent arterioles and own organ-specific effects (125). Fedele et al. showed that based on standard HF therapy, levosimendan could significantly increase GFR compared with placebo for CRS-1 patients with moderate renal impairment (126). Similar favorable results were also observed in Yilmaz et al.'s study. For CRS-1 patients requiring inotropic therapy, levosimendan could provide additional renal protective effects compared with dobutamine (127).

### Adenosine Antagonists

Adenosine is a nucleoside that is generated by phosphohydrolase of precursor molecules, including adenosine triphosphate and adenosine monophosphate. Adenosine is an important compound in renal hemodynamic regulation and can induce renal vasoconstriction and reduce GFR via activating adenosine A1 receptors expressed on afferent arteriole. The secretion of adenosine will dramatically increase in response to renal hypoxic and ischemic injuries (128, 129). Besides, it can also enhance water and sodium reabsorption by stimulating adenosine A1 receptors on the proximal tubules. Based on this evidence, for patients with AHF with renal dysfunction, adenosine A1 receptor antagonists are thought to be novel pharmacologic agents for their antagonistic effects of renal vasoconstriction and favorable effects of diuresis, natriuresis, and amelioration of renal infusion (130).

In Pilot Effects of Rolofylline, a New Adenosine A1 Receptor Antagonist on Symptoms, Renal Function, and Outcomes in Patients with Acute Heart Failure (PROTECT) study, a total of 301 patients with AHF with impaired kidney function (estimated creatinine clearance between 20 and 80 ml/min) were enrolled and were randomized to the rolofylline (a type of adenosine A1 receptor antagonists) therapy group with different dosage administration and the placebo group. The serum creatinine concentration increased in patients on placebo therapy and remained stable or tended to decrease in those receiving rolofylline therapy. Besides, treatment with 30 mg rolofylline was associated with reduced 60-day mortality or readmission for cardiovascular or renal causes. The kidney protective effects of rolofylline were validated in this trial (131).

Based on the phenomenon that rolofylline has a renal protective effect, the Placebo-Controlled Randomized Study of the Selective A1 Adenosine Receptor Antagonist Rolofylline for Patients Hospitalized With Acute Decompensated Heart Failure and Volume Overload to Assess Treatment Effect on Congestion and Renal Function (PROTECT) trial, which recruited more participants (amount to 2,033 patients), was designed and conducted to provide additional details about the adenosine A1 receptor antagonist therapy. Interestingly, inconsistent with the prior results, there were no statistically significant differences in short-term renal function changes and persistent WRF incidence between the two groups, which indicated no renal protective effect of rolofylline in patients with CRS-1 (132). No clear renal protective effect of rolofylline was also found in a multicenter, randomized, double-blind, Placebo-Controlled Study of the Effects of KW-3902 Injectable Emulsion on Heart Failure Signs and Symptoms, Diuresis, Renal Function, and Clinical Outcomes in Subjects Hospitalized With Worsening Renal Function and Heart Failure Requiring Intravenous Therapy (REACH UP) study (133). Until now, for rolofylline, the beneficial effect of preservation of renal function is imprecise.

### Ultrafiltration

Ultrafiltration is a mechanically therapeutic method to remove excess fluid through a semipermeable membrane by the actuation of transmembrane pressure gradient (134). Current studies and clinical practice guidelines have not recommended ultrafiltration as a routine strategy to tackle over-congestion in CRS-1, even in those who have a poor response to diuretics. However, for patients with refractory volume overload, obstinate DR, renal deficiency, severe hyperkalemia, and acidosis, ultrafiltration is recommended and can serve as an effective therapeutic regimen (122).

In the Aquapheresis vs. Intravenous Diuretics and Hospitalization for Heart Failure (AVOID-HF) trial, compared with patients with AHF on adjustable intravenous loop diuretics therapy, those receiving adjustable ultrafiltration therapy had a better decongestive effect and reduced risk of recurrent HF and cardiovascular events within 90 days of discharge from the index hospitalization. It is remarkable that adjustable ultrafiltration therapy would not worsen renal function (135). In previous randomized controlled trials, including the Early ultrafiltration in patients with decompensated heart failure and diuretic resistance (EUPHORIA) trial and the Ultrafiltration vs. Intravenous Diuretics for Patients Hospitalized for Acute Decompensated Congestive Heart Failure (UNLOAD) trial, no WRF was detected in patients with AHF receiving ultrafiltration therapy (136, 137). Suspicion of renal safety of ultrafiltration is proposed in the Cardiorenal Rescue Study in Acute Decompensated Heart Failure (CARRESS-HF) trial which indicated the correlation of ultrafiltration therapy and increased risk of WRF in patients with acute CRS (138). The opposite results of the CARRESS-HF trial were further evaluated. Although the deterioration of renal function in the ultrafiltration group was statistically significant but may not be clinically significant. Besides, the serum creatinine concentration (0.23 ± 0.70 mg/dL) did not reach the acknowledged criteria of WRF (0.30–0.50 mg/dL or greater), which has been described above (134).

## Conclusion

CRS-1 is a major clinical problem and WRF predicts a poor prognosis. Despite no fully explicit mechanism, multiple factors, including hemodynamic imbalance, neurohormonal activation, sympathetic activity, pharmacological interventions, inflammation along with oxidative stress, are involved in pathogenesis. Numerous biomarkers have a good performance in the early identification of WRF in CRS-1 while some are not so excellent. Effective treatment strategies with good renal safety have been explored and further studies should be conducted.

## Author Contributions

All authors listed have made a substantial, direct, and intellectual contribution to the work and approved it for publication.

## Funding

This study was supported by the National Natural Science Foundation of China (81873516, 81873522, and 81900444), the National Key Research and Development Program of Shandong Province (2017YFC1308303), the Shandong Provincial Natural Science Foundation of China (ZR2019PH030), and the Clinical Research Center of Shandong University (No. 2020SDUCRCA009).

## Conflict of Interest

The authors declare that the research was conducted in the absence of any commercial or financial relationships that could be construed as a potential conflict of interest.

## Publisher's Note

All claims expressed in this article are solely those of the authors and do not necessarily represent those of their affiliated organizations, or those of the publisher, the editors and the reviewers. Any product that may be evaluated in this article, or claim that may be made by its manufacturer, is not guaranteed or endorsed by the publisher.
